# Recycling of Date Pits Into a Green Adsorbent for Removal of Heavy Metals: A Fractional Factorial Design-Based Approach

**DOI:** 10.3389/fchem.2019.00552

**Published:** 2019-08-13

**Authors:** Khalid Al-Saad, Marwa El-Azazy, Ahmed A. Issa, Asma Al-Yafie, Ahmed S. El-Shafie, Maetha Al-Sulaiti, Basem Shomar

**Affiliations:** ^1^Department of Chemistry and Earth Sciences, College of Arts and Sciences, Qatar University, Doha, Qatar; ^2^Qatar Environment and Energy Research Institute (QEERI), Hamad Bin Khalifa University, Doha, Qatar

**Keywords:** dates' byproduct, heavy metals, fractional factorial design, kinetics, equilibrium

## Abstract

Date pits (DPs) have been recycled into a low-cost adsorbent for removing of selected heavy metals (HMs) from artificially contaminated aqueous solutions. Adsorption of targeted HMs, both by raw date pits (RDP) and burnt date pits (BDP) was tested. Results showed that BDP is more efficient as an adsorbent and mostly adsorbing Cu(II). A novel approach; fractional factorial design (2^*k*−*p*^ – FrFD) was used to build the experimental pattern of this study. The effects of four factors on the maximum percentage (%) of removal (Y) were considered; pH, adsorbent dose (AD), heavy metal concentration (HMC), and contact time (CT). Statistically significant variables were detected using Pareto chart of standardized effects, normal and half-normal plots together with analysis of variance (ANOVA) at 95.0 confidence intervals (CI). Optimizing (*maximizing*) the percentage (%) removal of Cu(II) by BDP, was performed using optimization plots. Results showed that the factors: pH and adsorbent dose (AD) affect the response positively. Scanning electron microscopy (SEM) was used to study the surface morphology of both adsorbents while fourier-transform infrared spectroscopy (FTIR) was employed to get an idea on the functional groups on the surface and hence the adsorption mechanism. Raman spectroscopy was used to characterize the prepared adsorbents before and after adsorption of Cu(II). Equilibrium studies show that the adsorption behavior differs according to the equilibrium concentration. In general, it follows Langmuir isotherm up to 155 ppm, then Freundlich isotherm. Free energy of adsorption (Δ*G*_ad_) is −28.07 kJ/mole, when equilibrium concentration is below 155 ppm, so the adsorption process is spontaneous, while (Δ*G*_ad_) equals +17.89 kJ/mole above 155 ppm, implying that the process is non-spontaneous. Furthermore, the adsorption process is a mixture of physisorption and chemisorption processes, which could be endothermic or exothermic reactions. The adsorption kinetics were described using a second order model.

## Introduction

Release of heavy metals (HMs) into aquatic bodies by industrial activities and other sources, e.g., mining, acid rain, agricultural waste, etc. represent a global challenge. HMs and other emerging contaminants are posing a serious influence on the environment and human health (Goel et al., 2005; Gupta et al., 2018).

The increasing flux of HMs into aquatic environments and the properties of HMs (toxicity, degradation rates, accumulation, uptake, bioavailability, etc.) calls for strict rules and action plans for monitoring, detoxification methodologies, and treatment technologies to keep their concentrations within the permissible levels. Previous studies showed that copper [Cu(II)], and though being an indispensable element for the human beings, may cause brain and liver damage, hemolysis, anemia, convulsions, and other sever symptoms, that would finally end with death (Chowdhury et al., 2016; Gupta et al., 2018). As per the WHO International Standards for Drinking Water, allowable concentration should not exceed 2 mg/L (Copper, 1998; WHO, 2003).

Numerous technologies are being applied for wastewater treatment; primary, secondary, tertiary, and advanced. Processes include sedimentation, coagulation/co-precipitation, oxidation/reduction, extraction, reverse osmosis (RO), electrochemical treatment, ion-exchange, filtration, etc. Yet, some of these procedures, though being widely used, are of limited application either due to high cost or low efficacy. Others are tedious and need pre/post treatments (Fu and Wang, 2011; Norton-Brandão et al., 2013).

Adsorption, and in contrary, is an uncomplicated, economical, and a sediment-free process. Popular adsorbents include activated carbon, alumina and silica gels, chitosan, zeolites, and ion-exchange resins. By and large, a “*standard”* adsorbent should have certain features, such as: availability and accessibility, cost-effectiveness, ease of use, regeneration, versatility, selectivity, stability, and high surface area (Leung et al., 2000; Coelho et al., 2014; Deliyanni Eleni et al., 2015).

Agricultural wastes possessing these *standard* properties become a smart approach to remove HMs. Recycling of agricultural wastes, which represent a burden on the ecosystem, is becoming a target for lots of investigations (Leung et al., 2000; Alslaibi et al., 2013; Coelho et al., 2014; Deliyanni Eleni et al., 2015; Ali et al., 2016; Burakov et al., 2017). Date pits (DPs), and in Qatar as a palm-growing country, are among the most copiously available byproducts of dates. With an annual production of 8,460,443 metric tons of dates, around 960 thousand tons of pits are discarded. The physico-chemical structure of DPs, as well as being abundantly available at a low-cost, are the main factors in choosing pits as an *ideal* adsorbent for removal of water pollutants. Both RDP and BDP were used as adsorbents. BDP as a carbonaceous biomass possess notable features such as larger surface area, well-developed pore structure, and hence better adsorption capability compared to RDP (Rahman et al., 2007; Hilal et al., 2012; FAO, 2013; Samra et al., 2014).

Improving the adsorption efficiency and the performance of the adsorbent in general is the task undertaken in many investigations. The common pathway to have such a control is by managing the variables contributing to the adsorption process. These variables include basically: pH, adsorbent dose (AD), heavy metal concentration (HMC) and contact time (CT) (Sahu et al., 2009; Bisht et al., 2017). The common approach in most investigations is usually univariate based. In other words, one factor at a time is being inspected. In addition to being time and effort consuming, method *greenness* is lost. That is because of consumption of chemicals, resources, and hence production of waste, through the large number of experiments performed. Additionally, the data obtained cannot be treated with a high degree of certainty (Zhu et al., 2014; Elazazy, 2015, 2017; Elazazy et al., 2016).

Eco-design of a *standard* adsorbent can be approached by having an experimental design. Factorial design, and as a multivariate approach, overcomes the difficulties encountered with the univariate analysis. Moreover, a full insight on the process under consideration can be drawn with the best factorial limits, and their interactions (Zhu et al., 2014; Elazazy, 2015, 2017; Elazazy et al., 2016; El-Azazy et al., 2019a,b). A fractional factorial design (2^*k*−*p*^ – FrFD) will be the design of choice in the current treatise (Antony, 2003; Abdel-Ghani et al., 2009; Hibbert, 2013; Elazazy, 2015, 2017; Elazazy et al., 2016). Another plus of the current approach compared to the previous reports is that the process we are following to prepare the carbonaceous mass depends mainly on thermal activation of DPs without use of chemicals. Table 1 shows a comparison between reported investigations on usage of DPs and the current approach.

**Table 1:** A comparison between the performances of DPs prepared in the current approach and as reported in literature.

Adsorbent (DPs)^*^	**Modification Method**	**Analytical approach used**	**HM removed**	**BET surface area and pore volume**	**Adsorption Capacity (mg/g)**	**Adsorption/ removal (%)**	**References**
Untreated date pits	Samples were washed, dried for 2 h at 125°C, crushed and then sieved with size (25–63 μm)	Univariate analysis	Pb (II)	ND	2.89 mg/g	95%	Samra et al., 2014
Raw and activated date pits;RDPADP	Date pits were washed and dried at 80°C for 2 h, crushed, grinded and sieved in a sieve series 60-mesh. For ADP; powder was mixed with 85% phosphoric acid in weight ratio 1:3 and heated to 160°C	Univariate analysis	Cu (II) Cd (II)	ND	**Cu(II)** RDP: 7.40 mg/g ADP: 33.44mg/g **Cd(II)** RDP: 6.02 mg/g ADP: 17.24mg/g	96.67%	Hilal et al., 2012
RDPPDP_100, 24_KOH-DPACDPAC	The seed powder was washed and dried at 120°C for 8 h. Then the powder was soaked with 85% H_3_PO_4_ with ratio of 1:2.5. After 12 h. of impregnation, the filtered date pits powder was subjected to carbonization in a muffle furnace at 650°C for 120 min	Univariate analysis	Pb (II)	**RDPP:** 0.027 m^2^/g, and 0.255 cm^3^/g **DPAC:** 316.9 m^2^/g, and 1.167 cm^3^/g	10.53 mg/g 31.69 mg/g 55.27 mg/g 115.83 mg/g	99.4%	Krishnamoorthy et al., 2019
Activated carbon of date pits;AC1AC2AC3	**AC1:** DP, Steam/N_2_, 1 h at 700°C. **AC2:** Date pits, steam, calcium acetate, 1 h at 700°C. **AC3:** Date pits, pure steam, 1 h at 800°C	Univariate analysis	Co (II) Fe (III) Pb (II) Zn (II)	**AC1:** 0.0188 m^2^/g, and 0.095 cm^3^/g **AC2:** 0.029 m^2^/g, and 0.248 cm^3^/g **AC3:** 0.0702 m^2^/g, and 0.321 cm^3^/g	**(AC3)** **Co (II):** 1,317 mg/g **Fe (III):** 1,555 mg/g **Pb (II):** 1,261 mg/g **Zn (II):** 1,594 mg/g	95%	Awwad et al., 2013
Untreated date stones (D.S)	Dates were washed with water, dried for 24 h at 105°C, crushed and sieved with size (1 mm)	Univariate analysis	Cr (VI)	1.2 m^2^/g, and 0.02 cm^3^/g	**Free pH:** 18.2 mg/g **pH (2):** 70 mg/g	ND	Khelaifia et al., 2016
Untreated date pitsRDP	Dates were washed, dried for 24 h at 70°C, crushed and sieved with size (250 μm)	Univariate analysis	Au (III)	**M.B. adsorption method:** 285 m^2^/g	**With 0.5 M HCl:** 78 mg/g	90%	Al-Saidi, 2016
**Current approach**							
RDP	Please see the experimental section	Multivariate analysis	Cu(II)	**RPP:** 2.72 m^2^/g and 0.007987 cm^3^/g	ND 4.036 mg/g	47.65%	
BDP	Please see the experimental section			**BPP:** 158.1 m^2^/g and 0.136163 cm^3^/g		98.51%	

* *Adsorbent (DPs) given names and abbreviations are as mentioned in the corresponding reference. ND, not determined*.

The aim of this work is to develop an adsorbent from date pits (DPs) (both raw and burnt) with a superior competency in removing selected HMs from aqueous solutions. The design will be structured through three phases; screening implementing the fractional factorial design, tuning using the response optimizer, and verification by operating analysis of variance (ANOVA) after each design stage. The adsorption mechanism will be explained following characterization of the adsorbent surface using Brunauer-Emmett-Teller (BET) surface area and pore size determination, fourier transform infrared spectroscopy (FTIR), Raman spectroscopy, and scanning electron microscopy (SEM). Moreover, thermal characterization of the adsorbent using thermal gravimetric analysis (TGA) will help understanding the physico-chemical properties of DPs. Prepared sorbent samples were further used for equilibrium and kinetics studies to investigate adsorption of Cu(II) on BDP.

## Experimental

### Materials

Analytical grade reagents were used throughout the experiments. Dates were collected from local farms in Qatar. All samples were prepared and diluted using ultrapure water (18.2 MΩ). A heavy metal mixture (1,000 ppm mixture of Cd, Co, Cu, Fe, La, Ni, and Pb) was prepared for ICP-OES investigations. Copper (500 ppm) stock solution has been prepared using copper nitrate trihydrate (Cu(NO_3_)_2_.3H_2_O, Fluka, USA). Adjustment of pH was done using small aliquots of 2% NaOH and 2% HCl. All glassware was soaked overnight in 5% nitric acid and then washed with deionized water.

### Instrumentation

A Waring Commercial blender was used to crush clean DPs. A drying oven (Memmert ULE 700, Germany) and a furnace (Thermolyne, 48000) were used to dry and burn clean crushed DPs. Both RDP and BDP were characterized using scanning electron microscopy (SEM, FEI, Quanta 200, USA) and energy dispersive X-ray spectroscopy (EDX). Fourier Transform Infrared Radiation (FTIR, Bruker Alpha, USA) was used to identify the functional groups on the adsorbent surface. Spectra were obtained in the range of 400–4,000 cm^−1^ with a resolution factor of 4 cm^−1^. Thermal gravimetric analyzer (TGA, PerkinElmer-TGA400) was used to study the thermal stability pattern of the adsorbent at a temperature range of 50–800°C. The Raman spectrum before and after adsorption of Cu(II) was acquired in the range from 50 to 3,500 cm^−1^ using a DXR™ 2 Raman microscope (Thermo, USA), with a laser beam at 532 nm as excitation source and 10 mW of power. Inductively coupled plasma optical emission spectroscopy (ICP-OES, PerkinElmer - Optima 7300 DV); was used to test the adsorption capacity of DPs on a multi-element solution.

Specific element measurements were performed using an Atomic Absorption Spectrophotometer (AAS, Shimadzu 6800). An ultrasonic shaker (Branson 2800- Bransonic) was used to shake the samples for the specified contact time. A Thermo Scientific centrifuge (SL8) was used to centrifuge the samples after shaking. For filtration; Whatman syringe filters (0.45 μm pore size) were employed. For surface area analysis, a Micromeritics ASAP 2020 Accelerated Surface Area and Porosimetry System was employed. Samples were first processed (degassed) and then N_2_ adsorption-desorption was conducted. Based on the N_2_ isotherms at 77 K and using the Brunauer Emmett-Teller (BET) equation, surface area was estimated. Pore volume was determined using the t-plots and the Barrett–Joyner–Halenda (BJH) equation.

### Software

Minitab®17 software (Minitab Inc., State College, Pennsylvania, USA) has been purchased to construct the experimental design.

### Procedure

#### Adsorbent Preparation

Dates, collected from local farms in Qatar, were washed and pits were manually removed. Date pits (DPs) were washed with distilled water 4–6 times at room temperature and then with hot distilled water to remove any dirt. DPs were then dried in the oven at 100°C in the morning and the temperature was then reduced to 60°C at night for 3 successive days. Dried DPs were then crushed and grinded into a fine powder and passed through sieves of different pore sizes. Crushed DPs were divided into two portions; one half was burnt (burnt date pits, char, BDP) while the other half was tested as it is (raw date pits, RDP). For burning (thermal activation), an amount of 10 g of crushed DPs was placed in a clean dry crucible, covered with crucible cover and burnt in a furnace at 500°C for 30 min. The crucible was then allowed to cool down and the ash was removed and collected in glass bottles and kept in the desiccator.

#### Assessment of Adsorption Capabilities of RDP and BDP

Screening of adsorption capability of both RDP and BDP was performed using 1 ppm multi-element mixture of Cd(II), Co(II), Cu(II), La(III), Ni(II), and Pb(II). Two sets of 15 mL centrifuge tubes were prepared, one for RDP and the other is for BDP. An amount of 0.1–0.5 g of the adsorbent and a volume of 10.0 mL of the HMs' blend were mixed into the centrifuge tube. Blanks were similarly prepared and then the tubes centrifuged at 4,200 rpm for 30 min. The supernatant was filtered using a syringe filter into a new array of 15 mL bottles. All filtered solutions were analyzed using ICP-OES. Comparison was conducted in terms of % of the HM adsorbed, Y, calculated using Equation 1. where *X*_*i*_ and *X*_*f*_ are the initial and final concentrations of the HM.

(1) $$\begin{array}{r} \%\text{ of HM adsorbed }\left(\text{Y}\right)=\frac{X_{i}-X_{f}}{X_{i}}\times 100\% \end{array}$$

#### Construction of the Design Matrix: 2^4−1^ FrFD

A fractional factorial design (2^*k*−*p*^- FrFD) has been formulated employing Minitab®17 software, where *k* is the number of factors and *p* is the size of the fraction used from a full factorial design (FFD). For the 4 investigated factors, 12 runs were generated, and 4 center points were added. Experiments were conducted in two blocks confounded with the two-way interactions. One response was measured, Y, % of HM removed by the adsorbent. The defining relation was given as I = ABCD, with the design generators D = ABC and block generators are AB. The alias structure is given as: I + ABCD, Blk = AB + CD, A + BCD, B + ACD, C + ABD, D + ABC, AC + BD, and AD + BC. Response optimization was performed using a response optimizer implementing the desirability plots. Studied variables and investigated limits are shown in Table 2. Detailed matrix for the conducted runs is shown in Table 3. It is noteworthy to mention that after completion of the CT specified in Table 3, each tube was centrifuged at 4,500 rpm for 30 min., the supernatant was then filtered through a Whatman syringe filter and then analyzed using FAAS.

**Table 2:** Proposed fractional factorial design for coded variables.

**Variables and their codes**	**Lower domain (−1)**	**Mid domain (Ct.Pt., 0)**	**Upper domain (+1)**
pH (pH, **A**, pH unit)	3	6	9
Adsorbent dose (AD, **B**, g/50 mL)	0.1	0.35	0.6
Heavy metal concentration (HMC, **C**, ppm)	1	5.5	10
Contact time (CT, **D**, min.)	1	60.5	120
Response “Y”	Maximum % of HM removal		

*Factor domains are represented as (−1, low), (0, central point), and (1, high). Selected HM is Cu(II)*.

**Table 3:** Experimental pattern for coded variables using 2^4−1^-FFD.

**Run**	**Coded levels of variables**				**Response (Y, %)**		
	**A^**a**^**	**B^**b**^**	**C^**c**^**	**D^**d**^**	^*^Obs.	^**^Pred.	^***^Pred.
1	−1	+1	+1	−1	71.15	67.85	74.55
2	0	0	0	0	93.00	86.16	86.27
3	+1	−1	−1	+1	92.22	88.92	91.94
4	+1	−1	+1	−1	99.61	96.31	99.87
5	0	0	0	0	85.93	86.16	86.27
6	−1	+1	−1	+1	87.03	83.72	84.13
7	+1	+1	+1	+1	99.16	102.5	98.16
8	−1	−1	−1	−1	18.43	21.74	31.33
9	−1	−1	+1	+1	10.20	13.51	^****^
10	0	0	0	0	85.12	86.16	86.27
11	0	0	0	0	80.59	86.16	86.27
12	+1	+1	−1	−1	88.98	92.30	90.08

*Response is shown as observed and predicted % of removal of Cu (II) using BDP*.

*A, B, C, and D^a^ are as defined in Table 1*.

* *Obs.: experimental values*.

** Pred.: predicted values before response transformation (factorial interactions up to 4th order was considered, prediction is averaged over blocks): Y = 86.16 + 24.14 pH + 15.73 AD – 0.82 HMC + 1.30 CT – 13.35 pH^*^AD+ 5.21 pH^*^HMC – 0.61 pH^*^CT – 15.31 pH^*^AD^*^HMC^*^CT.

*** Pred.: predicted values after response transformation (factorial interactions up to 4th order was considered): Y∧2= 7,443 + 2,887 pH + 1,439 AD – 1,609 pH^*^AD + 760 pH^*^HMC – 1,286 pH^*^AD^*^HMC^*^CT.

**** *Value was not detected by the model*.

#### Equilibrium and Kinetics Studies

A stock solution of 500 ppm Cu(II) was prepared from Cu(NO_3_)_2_.3H_2_O without adjusting the pH. Further dilutions of the stock solution; 5,10, 20, 40, 60, 80, 100, 150, 200, 300, and 400 ppm were prepared. Equal quantities of BDP (0.1 g) were added to 15 mL of the previously prepared solutions. Five different weights (0.05–0.35 g) of BDP were added to 13 mL of 80 ppm. All samples were then placed in shaker for 30 h. The kinetic study was conducted using three portions of 200 mL Cu(II) solution (30, 100, and 300 ppm) and ~ 1.5 g of BDP with magnetic stirrer. In case of 30 and 300 ppm, the sampling time was around 3 min. for 30 min., while in case of 100 ppm, it was per hour for 30 h, to confirm the equilibrium concentration. The case of 30 ppm will be discussed in the kinetics section since concentration of the adsorbate was very low compared to the adsorbent.

## Results and Discussion

### Screening and Optimization of Experimental Conditions

The purpose of the current investigation is to structure an economical adsorbent by recycling of locally available materials; DPs, that would represent a burden if not appropriately reused. Moreover, the proposed plan involves boosting of the adsorption efficiency of the DPs-derived adsorbent through a sound design. Selecting BDP as a candidate adsorbent was followed by constructing an experimental design for screening of the variables affecting the adsorption process. A multivariate analysis-based strategy (four variables and a single response); 2^4−*1*^ FrFD was exploited to build a model adsorbent. A 2^*k*−*p*^ FrFD is generally used when a full factorial design (FFD) cannot be afforded, where the usage of a fraction index of one would generate half-number of runs. The proposed design would facilitate the description of both large effects as well as their interactions (Antony, 2003; Abdel-Ghani et al., 2009; Hibbert, 2013; Zhu et al., 2014; Elazazy, 2015, 2017). Design matrix and obtained response are shown in Table 3.

#### Quality Charts and ANOVA

**Figure 1:**
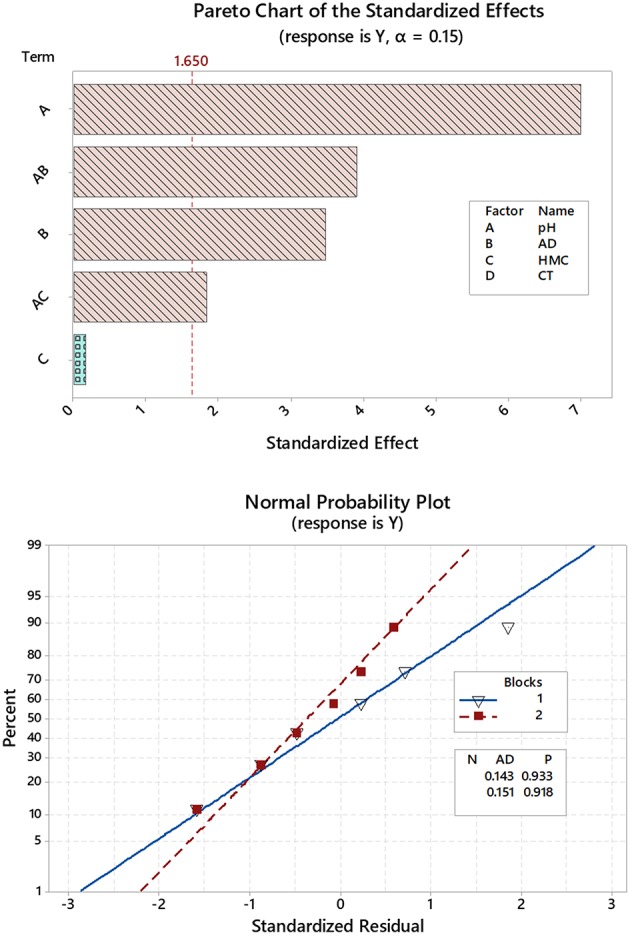
Pareto chart drawn after reisponse transformation (upper panel) and Normal probability plots where variables are grouped as blocks (lower panel).

Quality charts such as Pareto chart of standardized effects, Figure 1 serves to highlight the statistically significant variables, which and as shown are those factors beyond the reference line. From the chart, it can be concluded that linear factors such as pH (A) of the solution and AD (B) are statistically significant. Interactions like pH^*^AD (AB) was also effective and with a more weight compared to the AD (B) by itself. Identical outcomes were confirmed by plotting normal and half-normal plots of the effects (charts are not shown) as well as analysis of variance (ANOVA) (Box and Cox, 1964; Bruns et al., 2006; Vera Candioti et al., 2014). Better norm plots (Figure 1) were attained by stepwise regression combined with Box-Cox transformation (Box and Cox, 1964) and using the following formula:

(2) $$\begin{array}{r} \left(\text{Transformed response}\right)\;{\text{Y}}^{\text{′}}=\left({\text{Y}}_{\text{λ}}-1\right)\;/\lambda \;\left(\text{transformation factor}\right) \end{array}$$

Box-Cox plots at 95.0 confidence intervals (CI) showed a curve minimum at a λ value of 2. For stepwise regression, α value to add and remove was 0.15, and restoration of normality was indicated by *p*-value > 0.05 and better Anderson-Darling (AD) statistic (Anderson and Darling, 1954) in comparison to the non-transformed data. The regression formula in un-coded units obtained after these treatments and considering up to 2nd order interactions was as follows:

(3) $$\begin{array}{l} {\text{Y}}^{\wedge }2=-3,940+1,403\text{pH}+18,628\text{AD}-322\text{HMC}\\ \;-2,145{\text{pH}}^{*}\text{AD}+56.3{\text{pH}}^{*}\text{HMC}\\ \left[{\text{R}}^{2}=0.9301,{\text{R}}^{2}(\text{adj}.)=0.8718,{\text{R}}^{2}(\text{pred}.)=\;0.6610\right] \end{array}$$

Considering up to 4th order factorial interactions in the proposed model has generated the following equation:

(4) $$\begin{array}{l} {\text{Y}}^{\wedge }2=7,443+2,887\text{pH}+1,439\text{AD}-1,609{\text{pH}}^{*}\text{AD}\\ \;+760{\text{pH}}^{*}\text{HMC}-1,286{\text{pH}}^{*}{\text{AD}}^{*}{\text{HMC}}^{*}\text{CT}\\ \left[{\text{R}}^{2}=0.9675,{\text{R}}^{2}(\text{adj}.)=0.9404,{\text{R}}^{2}(\text{pred}.)=\;0.8783\right] \end{array}$$

Moreover, the verification step using ANOVA at 95.0 CI, Table 4A showed a better lack-of-fit value following the transformation.

**Table 4A:** Analysis of Variance (ANOVA) for the transformed response.

**Source**	DF^*^	Adj SS^*^	Adj MS^*^	***F*-Value**	***P*-Value**
**Model**	5	108623826	21724765	15.96	0.002
**Linear**	3	83297850	27765950	20.40	0.002
pH	1	66679206	66679206	49.00	0.000
AD	1	16575871	16575871	12.18	0.013
HMC^**^	***1***	***42774***	***42774***	***0.03***	***0.865***
**2-Way Interactions**	2	25325975	12662988	9.31	0.014
pH^*^AD	1	20705150	20705150	15.22	0.008
pH^*^HMC^**^	***1***	***4620825***	***4620825***	***3.40***	***0.115***
**Error**	6	8164686	1360781		
Curvature	1	4411474	4411474	5.88	0.060
***Lack-of-Fit***	***3***	***2671305***	***890435***	***1.65***	***0.400***
Pure error	2	1081907	540953		
**Total**	11	116788511			

*Only linear factors and 2-way interactions are considered*.

* *DF is degrees of freedom, SS is sum of squares and MS is mean of squares*.

** *Variables with p-value > 0.05 and lack-of-fit appear bold and italic*.

As a diagnostic tool, experimental values of Y were compared to the predicted values before and after transformation (Table 3). Comparison shows a good match between Y values obtained experimentally and those from the mathematical model specially after transformation. Capability of the new model to predict a new observation was confirmed by a better R^2^ (pred.) values as being perceived after transformation. For simplicity, only the interactions up to the 2nd order was considered.

In both Equations (3) and (4), findings of Pareto chart were confirmed. Yet, these equations show that both pH (A) and AD (B) have a positive influence on Y as indicated by the plus sign, compared to the negative impact of their interaction as well as that of HMC (C). In general, the developed model gives a comprehension of both linear and 2-way interactions in lights of student's *t*-test and Fisher's test values. While the *p*-value in ANOVA test shows the statistical significance of each coefficient, the t-values is a measure of both impact and pattern (+ or –) (Montgomery, 1991). Therefore, and as shown in Table 4B, the smaller the values of *p* (<0.05) and the higher the magnitude of *t*-value, the more the impact of the investigated term. As clear from the regression models as well as the *p*- and *t*- values in Table 4B, pH (A) would be of a higher (positive) significance compared to AD (B) for example.

**Table 4B:** Estimated effects, regression coefficients with corresponding *t*- and *P*- values for a transformed and optimized response.

**Term**	**Effect**	Coef.^*^	SE Coef.^*^	***t*-Value**	***P*-Value**	VIF^*^
**Transformed Response**						
Constant		6,586	337	19.56	0.000	
pH	5,774	2,887	412	7.00	0.000	1.00
AD	2,879	1,439	412	3.49	0.013	1.00
*HMC*	*146*	*73*	*412*	*0.18*	*0.865*	*1.00*
pH*AD	−3,218	−1,609	412	−3.90	0.008	1.00
pH*HMC	*1,520*	*760*	*412*	*1.84*	*0.115*	*1.00*
**Optimized Response**						
Constant		6,586	366	18.02	0.000	
pH	5,774	2,887	448	6.45	0.000	1.00
AD	2,879	1,439	448	3.22	0.012	1.00
pH*AD	−3,218	−1,609	448	−3.59	0.007	1.00

* *Coef., Coefficient; SE, Standard error; VIF, Variance inflation factor*.

** *Terms with p-value >0.05 appear italic*.

#### Response Surface Analysis and Contour Plots

**Figure 2:**
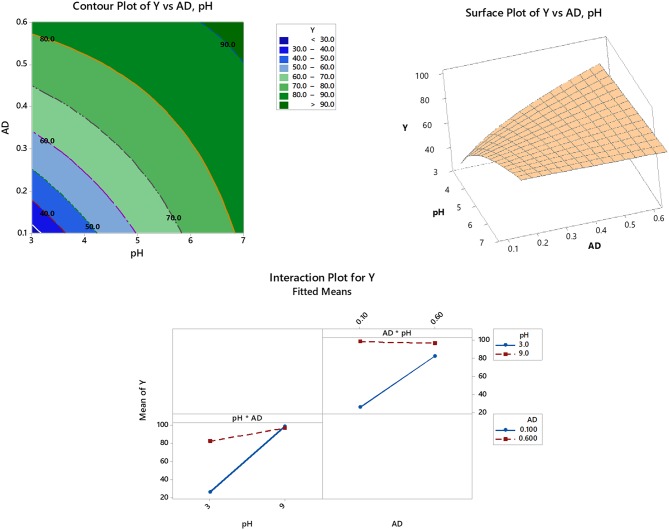
2D-Contour (upper left panel) and 3D-surface (upper right panel) plots of significant variable interactions and interaction plots of significant variables (model terms) after response optimization (lower panel).

The impact of interactions of significant variables and the response (% removal) can be shown through a 2D-view (contour plots) as well as the 3D-view (surface plots). As shown in Figure 2 (upper left panel), the inspected factorial interaction (pH and AD) is drawn on the X and Y axes, while the corresponding response is represented by the contour lines. As per the legend, the darkest green color denotes an area of maximum response, > 90% removal. The upper right panel of Figure 2 shows the same relationship but in the 3D format where the response is drawn on the Z-axis.

#### Response Optimization and Analysis of Variables' Impact

In this phase, and by consolidating the results of quality charts and ANOVA, variables with *p*-value > 0.05 were unconsidered. The following regression formula was obtained:

(5) $$\begin{array}{r} {\text{Y}}^{\wedge }2=-5,708+1,713\text{pH}+18,628\text{AD}-2,145\text{p}{\text{H}}^{*}\text{AD} \end{array}$$

A response optimizer tool was used for this purpose. The objective was to maximize the % of HM removal. Considering only model variables, maximum removal achieved was 98.19% with a desirability value of *d* = 0.9842, which is very close to the ideal desirability *d*= 1.0000 (Derringer and Suich, 1980; Myers and Montgomery, 2009). This high value of *d* implies that the used factorial blend (pH = 9 and AD = 0.1 g/50 mL) was in favor of the response.

Different factorial levels and combinations were employed to confirm the obtained desirability. As shown in Table 5, adsorption of Cu(II) increases as the value of pH increases. This observation is analogous to what has been reported in literature (Chen et al., 2011; Pellera et al., 2012; Kılıç et al., 2013). This could be attributed to the high [H^+^] at low pH, which in turn interferes with the adsorption of the HM on the target site. Having pH = 3 as an example, the % removal was the lowest among the reported responses at the other pH values (Table 5). Increasing the AD up to 0.6 g/50 mL has improved the response as well as the desirability. Yet, at higher pH values, the surface charge and hence the complexation performance of the adsorbent are greatly improved. This is not absolute! Where at pH≫6, the risk of having the metal hydroxide precipitating from the aqueous solution exists. Thus, it is not recommended to conduct the adsorption process (at least in our case) at a pH higher than 6 (Li et al., 2017; Mahdi et al., 2018). Having pH = 9 as an example, it can be noticed that there is no difference in the achieved % removal and the desirability with the increased AD. This finding implies that the HM might have been removed by precipitation rather than adsorption.

**Table 5:** Response and desirability values at different optimization conditions.

**Variable**	**Response (% removal)**	**Desirability**
**pH** **=** **3**		
AD = 0.1 g/50 mL	25.51	0.1712
AD = 0.6 g/50 mL	82.14	0.8046
**pH** **=** **6**		
AD = 0.1 g/50 mL	71.74	0.6883
AD = 0.6 g/50 mL	89.58	0.8879
**pH** **=** **9**		
AD = 0.1 g/50 mL	98.19	0.9842
AD = 0.6 g/50 mL	96.45	0.9647

*Only model variables; pH (A) and AD (B) were considered in the optimization phase*.

On the other hand, increasing the AD is usually associated with an enhanced removal efficiency. This might be attributed to an increased area of the adsorbent surface and consequently the removal efficiency (Esposito et al., 2001; Xie et al., 2015; Iftekhar et al., 2018). Keeping in mind that pH^*^AD interaction has a negative impact on the response as concluded from ANOVA, Table 4A and Figure 2 (lower panel) confirm this finding. As shown in the interaction plot, increasing the AD from 0.1 to 0.6 g/50 mL (upper right matrix) greatly enhances the adsorption at pH = 3, compared to almost no or negative effect at pH = 9. Similarly, increasing the pH from 3 to 9 has a higher impact on the removal efficiency at AD = 0.1 g/ 50 mL, compared to a lower influence at AD = 0.6 g/50 mL (lower left matrix).

Therefore, the optimum conditions that can be considered for the further experiments would be a pH = 6, and AD = 0.6 g/50 mL (The CT was kept at 55 min. and HMC of 5.5 ppm).

### Characterization

#### Fourier Transform Infrared Spectroscopic Analysis (FTIR)

As a part of comprehending the adsorption mechanism, FTIR was conducted for both RDP and BDP. FTIR serves as an *informative* tool to identify the functional groups on the surface of the adsorbent and how these groups are affected by the experimental conditions. RDP is a lignocellulosic material that consists mainly of cellulose, hemicellulose, lignin, and protein. Cellulose and hemicellulose are oxygen-rich constituents where a plenty of oxygen-containing functional groups such as hydroxyl, ether and carbonyl groups exist. Presence of these groups on the surface of RDP explains its capability to adsorb HMs (El-Hendawy, 2006).

**Figure 3:**
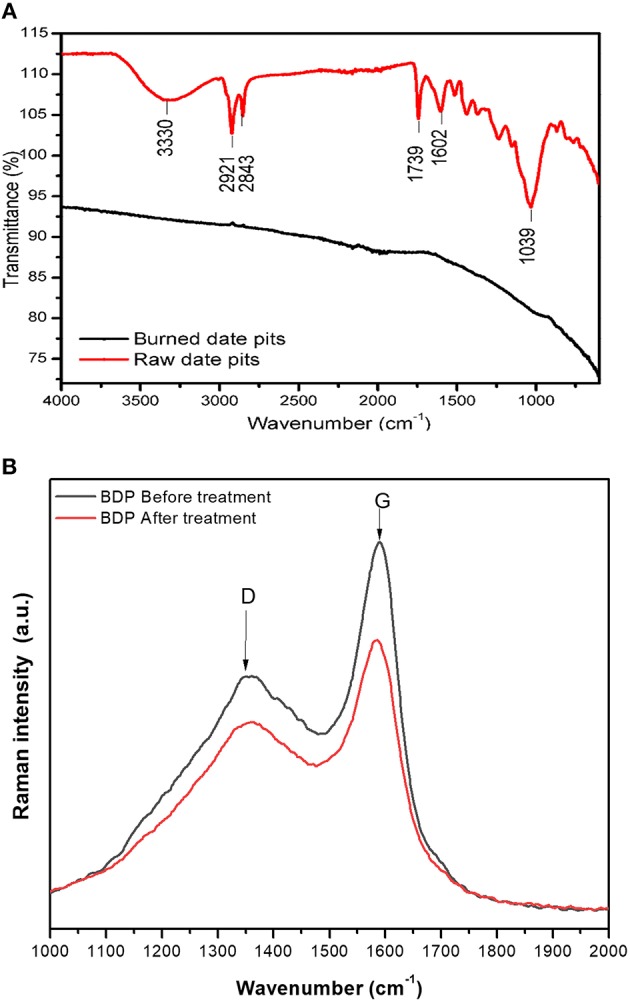
**(A)** Upper panel: FTIR of RDP (upper spectrum) and BDP (lower spectrum). **(B)** Lower panel: Raman spectra of BDP before (upper spectrum) and after (lower spectrum) adsorption of Cu(II).

As shown in Figure 3 (upper panel), a broad peak exists at 3,330 cm^−1^ which refers to the existence of -OH or -NH or both, while the two peaks at 2,921 and 2,843 cm^−1^ denote an aliphatic C-H stretching. Peaks at 1,739, 1,602, and 1,039 cm^−1^ indicate the presence of C=O (unconjugated carbonyl in xylan), C=C and C-O while the small peaks between them C=C and C-O referred to the bending peaks of methyl groups (Pandey and Pitman, 2003; El-Hendawy, 2006). On the other hand, the FTIR spectrum of BDP shows absence of the previously mentioned peaks, which indicates removing of the function groups and formation of pure carbonaceous residue.

Moreover, Raman spectroscopy was used to characterize BDP before and after adsorption of Cu(II) (Figure 3, lower panel). Obtained spectra of BDP show a clear D and G bands at approximately 1,348 cm^−1^ (D band) and 1,588 cm^−1^ (G band). These two bands; D and G are characteristic peaks in spectra of carbon materials. The D-, and G- bands pattern is very similar to that of graphene oxide (Stankovich et al., 2007). The D-band reflects the carbon lattice properties as defects and sizes, and not the chemical composition of the carbon materials. The location of the D-bands depends mainly on the wavelength of the laser beam, while the G-band represents the stretching of C-C in *sp*^2^ system (Childres et al., 2013). The G-band does not split, so there are no charged ions between the carbon layers, therefore we can conclude that Cu(II) did not diffuse inside the BDP grains. Moreover, the two peaks (D and G) are not present in the two spectra of the RDP (figures are not shown) confirming the formation of activated carbon after burning DPs. In addition, the intensity ratio I_D_:I_G_ is 0.63 and 0.69 for BDP before and after adsorption of Cu (II). This increase after adsorption could be a result of increasing defect states in the *sp*^2^ plane of carbon following the adsorption (Mondal et al., 2013; Shu et al., 2017).

#### BET and TGA Analyses

**Figure 4:**
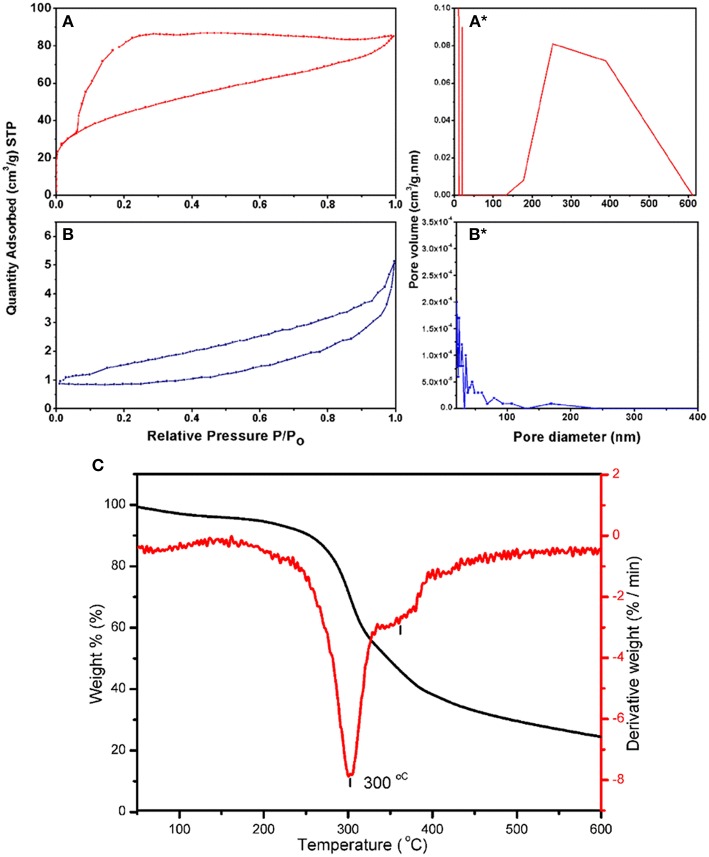
Nitrogen adsorption-desorption isotherms for **(A)** BDP and **(B)** RDP; pore diameters of (A^*^) BDP and (B^*^) RPD; and **(C)** TGA of the RDP.

Performance of the adsorbent is mainly dependent on the porosity of its surface and the area available to the HM during the adsorption process. The BET surface area and pore volume of both adsorbents were measured using N_2_ adsorption-desorption measurements and shown in Table 1. The BET surface area of BDP was at least 58 times greater than that of RDP. In addition, single point total pore volume for BDP was about 17 times higher than RDP. On the other hand, structural heterogeneity of the porous surface can be characterized by the pore size distribution (Conner et al., 1986; Altin et al., 1999; Chiang et al., 2001). According to the IUPAC classification of porous materials and referring to Figure 4A, BJH desorption average pore radius showed that RDP was mainly macroporous with few mesopores. On contrary, BDP was mainly mesoporous with fewer macropores.

Moreover, Figure 4 shows that RDP and BDP have types III and IV (H2) isotherms, respectively, indicating that BDP is porous and that as the concentration increases, adsorption of N_2_ gas occurs via multilayer adsorption followed by capillary condensation (gas filling). Furthermore, the H2 type indicates that the pores are of the inkbottle type, which are connected internally. This finding has been proved by analysis of SEM micrographs, as will be shown later in the corresponding section.

Thermal decomposition of RDP in the range of 30–800°C is shown in Figure 4C. From the thermogram; the dashed line represents TGA curve, while the solid line represents differential TGA curve (*d*TGA). A small peak was observed between 50 and 200°C, which might be attributed to the removed humidity and/or volatile compounds that represent ~8% of the sample. The main decomposition step was observed between 200 and 400°C. This step occurs in two stages; a sharp peak between 230 and 333°C, which corresponds to decomposition of 40.4% of the adsorbent sample (DPs), and the second, between ~330 and 380°C corresponding to decomposition of 19.6% of the adsorbent. The residual amount, 32.9% is the remaining adsorbent available for the adsorption process (Yang et al., 2007; Sanchez-Silva et al., 2012).

#### Scanning Electron Microscopy Analysis (SEM)

**Figure 5:**
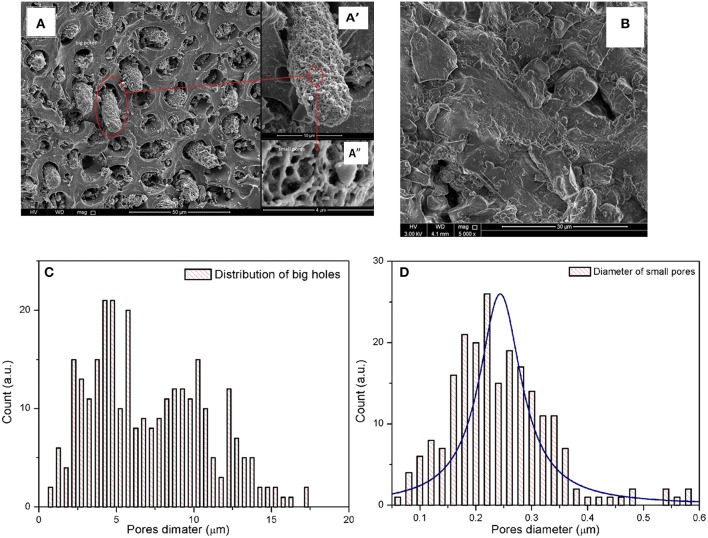
SEM micrographs of BDP at different scales **(A)** 50, **(A')** 10, and **(A”)** 4 μm, while **(B)** for RDP. The distribution of diameters are **(C)** big holes, and **(D)** macropores.

SEM was used to examine the surface structure of the raw and activated DPs. The SEM micrographs shown in Figure 8 revealed that the BDP (Figure 5A) was of higher porosity compared to the raw material (Figure 5B). The obtained data confirms the loss of all volatile and organic matter at 500°C and the formation of carbonaceous material with advanced pore structure (Hilal et al., 2012). These findings are in alignment with the BET surface area and TGA analyses. Comparing to the other thermally prepared carbonaceous materials, e.g., potato peels (El-Azazy et al., 2019a), the SEM micrographs of BDP show a completely different behavior, where the bulk keeps its structure with numerous holes possessing different sizes between 2 and 17 μm (Figures 5A,C) and are mainly formed by emitted hot gases. A column of highly porous carbonaceous material is formed during escalation of gases as shown in Figures 5A′,A″. The diameter distribution of those pores is between 0.1 and 0.4 μm (Figure 5D), which is aligned with the BET results. Moreover, the depth of those pores is not equal, because most of those pores have a different light intensity inside. In addition to this pore structure, a number of cracks (not shown) appears in the sample. Therefore, the SEM and BET indicate that the structure of BDP is solid blocks with holes and some cracks. The holes contain highly porous column inside, and gas filling occurs in those columns only. These data show that the thermal activation (treatment) of DPs is essential for enhancing the porosity and hence the adsorption process.

#### Energy Dispersive X-ray Spectroscopy Analysis (EDX)

**Figure 6:**
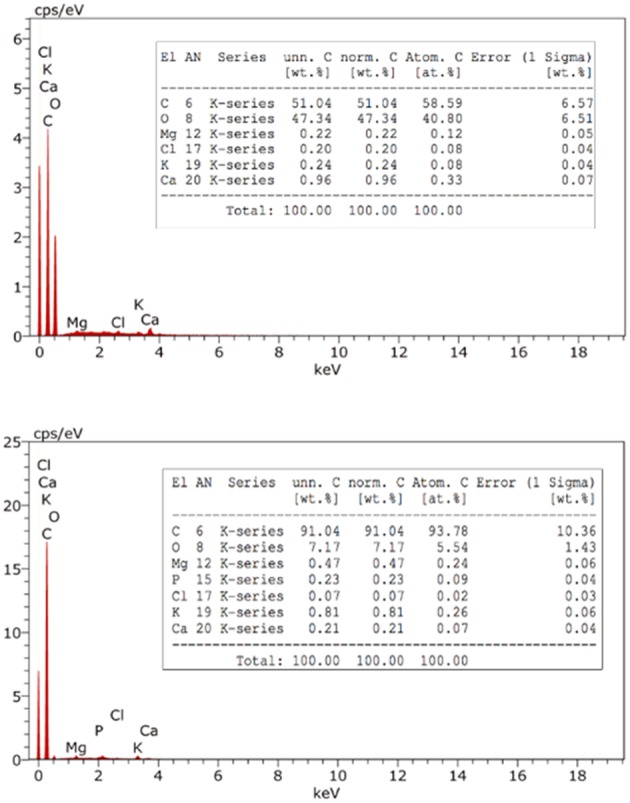
EDX analysis of RDP (upper panel) and BDP (lower panel).

The composition of BDP and RDP has been studied by EDX. The spectrum in Figure 6, upper panel shows that RDP consists basically of carbon and oxygen that constitute 51.04 and 47.34%, respectively. Elements such as magnesium, chloride, potassium, and calcium also exist but at a percentage <1%. Upon burning and as expected the % of carbon has increased to 91% indicating almost complete conversion of DPs into carbonaceous material.

#### RDP and BDP: Adsorption Mechanism and Evaluation of Adsorption Efficiency

Efficiencies of both adsorbents; RDP and BDP, were compared. Results show that for all elements tested (Cd, Co, Cu, La, Ni, Pb), the adsorption capacity of BDP was much higher compared to RDP. Among these elements, the most efficiently removed by RDP were Pb(II) (64.2%) and La(III) (55.9%). However, the most efficiently removed by BDP were Cu(II) (98.51%), Cd(II) (97.38%), and La(III) (95.10%). This notable capability of BDP can be attributed to the conversion of the raw material into a carbonaceous biomass physically by heating at elevated temperature. The formed carbonaceous material and as indicated by FTIR, BET, TGA, and SEM results has a larger surface area and pore volume compared to the untreated raw material. Moreover, distribution of pores and hence surface chemistry is better compared to unburnt material.

### Equilibrium and Kinetics Studies of Cu(II) Adsorption on BDP

The equilibrium and kinetic studies are very important in designing an efficient adsorbent. While the internal structure of the adsorbent cannot be properly revealed with traditional methods as SEM, it can be investigated using BET analysis. The maximum quantity (*q*_*m*_) which can be adsorbed, the pattern of adsorption on the surface of the biomass, the interaction between adsorbate and adsorbent's surface and whether it is chemisorption or physisorption, can be all determined using the adsorption isotherms. On the other hand, the rate of adsorption, thickness of the formed layer around the sorbent, and whether the reaction is controlled by diffusion or adsorption can be investigated employing kinetics study. In the next few sections, equilibrium (adsorption isotherms) and kinetics will be discussed.

#### Equilibrium Isotherms

**Figure 7:**
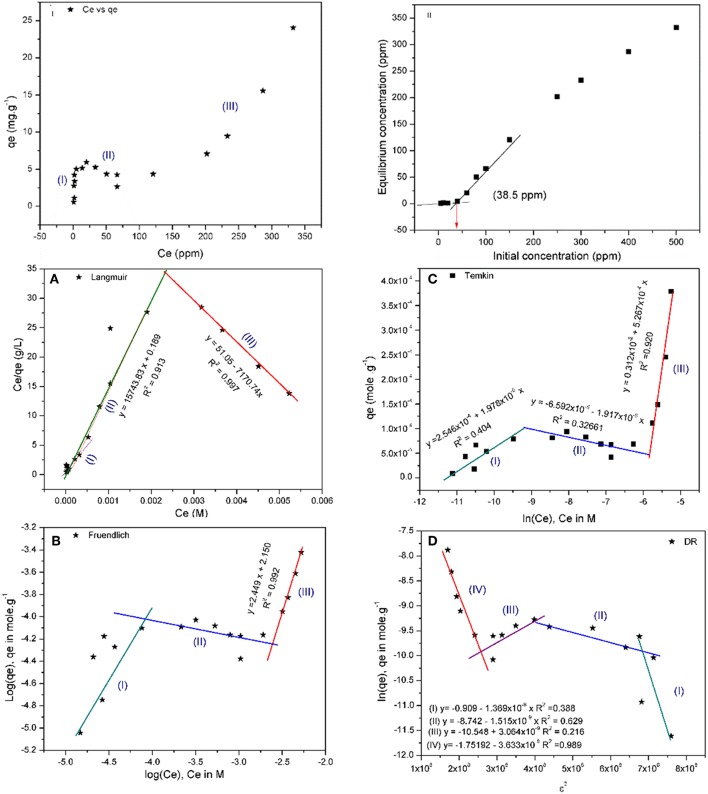
Relation between **(I)** *C*_*e*_ vs. *q*_*e*_ and **(II)** *C*_0_ and *C*_*e*_ for the adsorption of Cu(II) on BDP in addition to **(A)** Langmuir, **(B)** Freundlich, **(C)** Temkin, and **(D)** Dubinin-Radushkevich (DR) isotherms of Cu(II) adsorption on BDP.

Figure 7(I, II) shows that there are three different regions during the adsorption. Region I, starting at 0 to 4.8 ppm, in which the adsorption increases linearly with increasing the equilibrium concentration, followed by a pseudo- saturation region between 4.8 and 6.1 ppm before going to region II. In region II, the adsorption decreases linearly with increasing *C*_*e*_ from 6.1 to 65.7 ppm. Finally, region III, in which the adsorbed quantity (*q*_*e*_) increases exponentially with *C*_*e*_ and following the equation:

(6) $$\begin{array}{r} q_{e}=1.54\;e^{0.003\;C_{e}}\text{ with}{\;R}^{2}=0.993 \end{array}$$

All isotherms that have been used to fit the reported adsorption patterns emphasize the presence of those three regions, except in case of Langmuir isotherm. Explanation of this behavior is controversial. As per (Ryden et al., 1977), this behavior could be explained based on the presence of different types of sites on the adsorbent surface with variable free energy (Δ*G*_ad_). Others (Posner and Bowden, 1980), however, believe that the ligand exchange (OH^−^ or H_2_O) is the driving force for the adsorption process by changing the adsorption energy (Δ*G*_ad_) which can be defined as follows:

(7) $$\begin{array}{r} \Delta G_{ad}=RTln\left(a_{i}\right)-RTln\left(a_{is}\right)+\Delta G_{coul} \end{array}$$

Where *a*_*i*_ and *a*_*is*_ are the activity coefficient of adsorbate in solution, and on the sorbent surface, respectively, and Δ*G*_*coul*_ is the change in free energy due to electrostatic interaction between surface and ions. This finding was confirmed using a model with only one type of sites. The interpretation given herein with will adopt the second approach, where Cu(II) ions are attracted to the negatively charged surface until a certain limit at which the repulsion force between Cu(II) causes the formation of a layer around the sorbent. However, with the increase in [Cu(II)], the ionic strength of the solution increases leading to depletion of the boundary layer and *q*_*e*_ value increases again. Using Figure 7B to calculate the experimental value of maximum monolayer adsorbed quantity (*q*_*exp*_) and assuming that the adsorbed Cu(II) forms a monolayer first before going to equilibrium, then the calculated maximum adsorbed quantity is 5.5 mg/g, when the average value of used adsorbent is 0.105 g.

##### Langmuir isotherm

Figure 7A shows two segments. The first segment (I&II), where *C*_*e*_ is between 0 and 155 ppm, can be fitted to the traditional Langmuir isotherm. The Langmuirian zone could follow Langmuir multisite isotherm, with *q*_*m*_ = 4.036 mg/g which is close to the experimental value (5.5 mg/g), and *K*_*L*_ = 83.3 × 10^3^ (L. mole^−1^). Furthermore, the adsorption occurs spontaneously (indicated by negative ΔG_ad_), as shown in Table 6. The second segment (III), however, does not follow Langmuir isotherm because the *q*_*m*_ and *K*_*L*_ are negative values, besides the positive ΔG_ad_, implying a non-spontaneous adsorption.

**Table 6:** General and linarized equation of Langmuir, Freudlich, Temkin, Dubinin Radushkevich, and Hasley isothems, beside their parameters for adsorption of Cu(II) on BDP.

**Isotherm**	**Equations (generalized/linearized forms)**	**Curve segments**			
		**Parameters**	**(I)**	**(II)**	**(III)**
**Langmuir**	$q_{e}=\frac{q_{m}\;K_{L}\;C_{e}}{1-K_{L}\;C_{e}}$	*q*_*m*_(*mg*/*g*)	4.036	–	−0.885
		*K*_*L*_(L.mole^−1^)	83.3 × 10^3^	–	−1.4 × 10^3^
	$\frac{C_{e}}{q_{e}}=\;\frac{1}{q_{m}\;K_{L}}+\frac{C_{e}}{q_{m}}$	Δ*G*_*ad*_(kJ.mole^−1^)	−28.07	–	+17.89
		R^2^	0.913	–	0.997
**Freundlich**	$q_{e}=\;K_{F}C_{e}^{\frac{1}{n}}$	1/n	–	–	2.488
			–	–	141.3
	$\log\left(q_{e}\right)=\log\left(K_{F}\right)+\left(\frac{1}{n}\right)log\left(C_{e}\right)$	$K_{F}\left(mole/g\right){\left(L/mole\right)}^{1/n}$			
		R^2^	–	–	0.992
**Temkin**	$q_{e}=\;\frac{RT}{b_{T}}\;ln\left(A_{T}\;C_{e}\right)$	*b*_*T*_(*J*/*mole*)	20.41	−21.05	0.77
		*A*_*T*_ (L/mole)	7.4 × 10^12^	2.7 × 10^3^	8.4 × 10^5^
	$q_{e}=\;\frac{RT}{b_{T}}\ln\left(A_{T}\right)+\frac{RT}{b_{T}}\ln\left(C_{e}\right)$	R^2^	0.404	0.0.326	0.919
**DR**	$ln(q_{e})=\;\ln\left(q_{m}\right)-\;\beta \epsilon^{2}$	β	1.37 × 10^−8^	1.52 × 10^−9^	3.63 × 10^−8^
	$\epsilon =RT\left(1+\;\frac{1}{C_{e}}\right)$	*E*(kJ/mole)	6.04^**I**^	18.17^**II**^	3.71^**IV**^
		*q*_*s*_(*mg*.*g*)	7.83 × 10^3^	1.15 × 10^−4^	1.12 × 10^3^
	$E=\;\frac{1}{\sqrt{2\beta }}$	R^2^	0.388	0.629	0.949

*I, II, and IV refers to the energy of segments (I, II, and IV) of DR isotherm, respectively. While the data for segment (III)β, E, q_s_, and R^2^are 3.06 × 10^−9^,−12.77, 1.79 × 10^−6^, and 0.216, respectively*.

##### Freundlich isotherm

At *C*_*e*_ below 200 ppm, scattered points which cannot be fitted well were obtained, in contrast to data points obtained at *C*_*e*_> 200 ppm. Freundlich isotherm assumes that the adsorption is heterogeneous, energy of adsorption increases exponentially, and there is no saturation as assumed by Langmuir. Despite Freundlich model does not have a physicochemical meaning, its adjustable parameters (*1/n*) and *K*_*F*_ are indicative of the adsorption strength, heterogeneity and favorability. The results of Freundlich isotherms matches the findings of Langmuir confirming that the adsorption is heterogeneous and unfavorable due to (1/n) > 1 as shown in Figure 7B and Table 6.

##### Temkin isotherm

The three regions are clear in this model, especially region II which is well-fitted compared to the other isotherms (Figure 7C). Regions I and III are exothermic while region II is endothermic as shown by the value of *b*_*T*_ in Table 6.

##### Dubinin-Radushkevich (DR) isotherm

Figure 7D shows that the adsorption of Cu(II) on BDP occurs through four regions; three of which are as the other isotherms except segment (II) which can be divided into two subsegments. Energy values indicate that the adsorption of first and fourth steps is physisorption, while segemt II indicates chemisorption. The energy of segment III has a negative value, which could be explained considering the dissolution that occurs chemically as indicated in Table 6 (De et al., 2013; Kaveeshwar et al., 2018).

Though previous reports (Kinniburgh, 1986) showed that the adjustable parameters for Langmuir isotherm (*q*_*m*_ *and K*_*L*_) and other isotherms derived from the linearized forms of their equations did not give quantitative and accurate results compared to the non-linear least square regression (NLLS) of untreated experimental data; the linear form of those equations have been used in the current approach with the sake of simplicity. From the discussion of the above isotherms, it is clear that the adsorption follows Langmuir isotherm at low concentration (*C*_*e*_) up to 155 mg/L, then follows Freundlich isotherm at higher concentrations.

#### Kinetics Studies

The adsorption process can be represented by the following equation:

(8) $$\begin{array}{r} A+B\overset{k_{ad}}{\underset{k_{de}}{⇌}}AB \end{array}$$

Where A, B, AB are the adsorbate [Cu(II)], sorbent (BDP) and product of the adsorption process, respectively, while *k*_*ad*_ and *k*_*de*_ are the rate constant of adsorption and desorption, respectively. The rate of the reaction and equilibrium constant can be expressed as in the following equation:

(9) $$\begin{array}{r} Rate=k_{ad}{\left[A\right]}^{n}{\left[B\right]}^{m} \end{array}$$

Where the equilibrium constant $K=\;\frac{\left[AB\right]}{\left[A\right]\left[B\right]}$, and [A], [B], and [AB] are the concentrations of A, B, and AB; *n* and *m* are the reaction order with regard to A and B, respectively.

**Figure 8:**
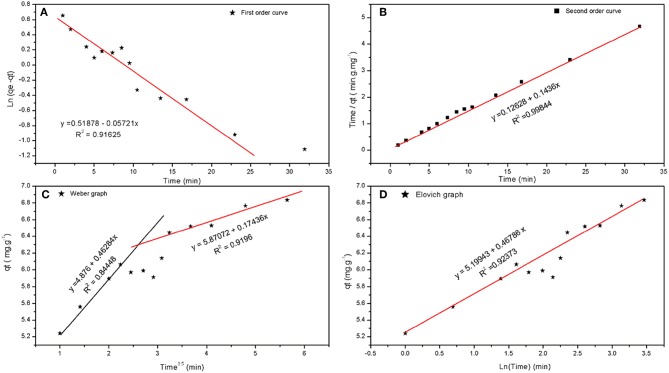
**(A)** First order, **(B)** second order, **(C)** intra particle diffusion (Weber), and **(D)** Elovich curves of adsorption of Cu(II) on BDP.

The kinetic models and their parameters are summarized in Table 7, in which the rate constants are shown, and the reaction seems to be pseudo-second order where a well-fitted curve of second order is shown in Figure 8B. The initial adsorption rate is extremely high compared to the desorption rate, where the ratio is 2.7 million times that calculated by Elovich equation as shown in Figure 8D. Moreover, Figure 8C shows that there are two diffusion rates with the reaction being a combination of adsorption and diffusion, and as the boundary layer increases the diffusion rate decreases.

**Table 7:** The kinetics study results corresponding to Figure 8.

**Models**	**Parameter**	**Value**
Pseudo-first order	*K_1_* (min^−1^)	0.057
ln(*q*_*e*_ − *q*_*t*_) = ln(*q*_*e*_) − *k*_1_*t*	*q_*e*_*(mg/g)	1.68
	R^2^	0.916
Pseudo-second order	K_2_ *(g.mg*^−1^*.min*^−1^*)*	2.6 × 10^−3^
$\frac{t}{q_{e}}=\;\frac{1}{k_{2}q_{e}^{2}}+\frac{1}{q_{e}t}$	*q_*e*_*(mg/g)	0.143
*Where K_2_ is rate constant (g.mg*^−1^*.min*^−1^*)*	R^2^	0.998
Elovich equation is *q*_*t*_ = β ln (αβ) + β ln(*t*) is used to predict the sorption mechanism, where q_t_ is adsorbed quantity at time t; while α and β are initial sorption concentration rate (mg.g^−1^.min^−1^), and desorption constant (g/mg), respectively	α B R^2^	1.43 × 10^5^ 0.468 0.923
Weber-Morris intraparticle diffusion model is used to study the formed layers around the adsorbent and rate-controlling step, which is expressed as *q*_*t*_ = $K_{I}t^{0.5}+C$, where K_I_ is intraparticle diffusion rate constant (mg.g^−1^.min^−0.5^), and C is boundary thickness effect.	K_I_ C R^2^	0.463 0.174 4.876 5.870 0.844 0.919

Form the Elovich and Weber-Morris graphs and depending on equilibrium studies, the adsorption mechanism can be explained as follows: at the beginning of the adsorption, the process is controlled by very fast adsorption of Cu(II) on the surface of carbon to form a positive layer. Subsequent to this layer, a negative layer (*from the counter ions*) is formed around the first layer. The Cu(II) ions then diffuse through this layer to reach the surface. As the thickness of this layer increases, the diffusion decrease and adsorption of Cu(II) on this negative layer increases.

## Conclusion

Date pits (DPs) have been used as green adsorbent to adsorb Cu(II) form artificially contaminated wastewater samples. Adsorption was studied implementing a fractional factorial design. The implemented design allowed studying variables impacting the adsorption with the minimum time and effort. Four factors were studied; pH, AD, HMC and CT. Obtained data showed that pH, AD, as well as their interactions significantly affect the removal efficiency of DPs. SEM micrographs revealed that the BDP is highly porous compared to RDP, due to formation of numerous big holes, which in turn contain columns of high porous carbonaceous materials. The diameters of big holes and macropores are ranged between 1 and 15, and 0.05 and 0.4 μm, respectively. This notification is confirmed by BET, where the surface area of RDP and BDP is 2.72 and 158.11 m^2^/g, respectively. An amount of 80% of the DPs is lost at 600°C, while the maximum degradation rate was at 300°C. The relation between *C*_*e*_ vs. *q*_*e*_ shows that there are three adsorption areas. Those three regions are shown by most of the used isotherms (Freundlich, Temkin, and DR). Equilibrium study shows that the adsorption obeys Langmuir isotherm at low concentrations while it follows the Freundlich isotherm at higher concentrations. Temkin isotherm shows that the adsorption is exothermic in segments (I and III), while it is endothermic in segment (II). Dubinin-Radushkevich (DR) shows four regions, segments (I and IV) are physisorption, while segment (II) is chemisorption. The negative value of segment (III) could be due to the desorption of Cu(II). The adsorption is second order reaction, which is controlled by both diffusion and adsorption mechanisms.

## Data Availability

All datasets generated for this study are included in the manuscript and/or the supplementary files.

## Author Contributions

KA-S: idea, surveying literature, supervision of student, and revision of manuscript. ME-A: experimental design, analysis of results, writing manuscript, and supervising student. AI: working on ICP, AAS, TGA, EDX, conducting kinetics and thermodynamics studies, and writing this section. AA-Y: performed the experiments in the design. AE-S: BET and SEM analysis, TGA, ICP, AAS, and put the manuscript in the journal format. MA-S: FTIR, SEM analyses, and supervising students. BS: revising manuscript.

### Conflict of Interest Statement

The authors declare that the research was conducted in the absence of any commercial or financial relationships that could be construed as a potential conflict of interest.
